# Commentary on muscle dysmorphia as an addiction: A response to Grant (2015) and Nieuwoudt (2015)

**DOI:** 10.1556/JBA.4.2015.1.4

**Published:** 2015-03-18

**Authors:** MARK D. GRIFFITHS, ANDREW C. FOSTER, GILLIAN W. SHORTER

**Affiliations:** ^1^International Gaming Research Unit, Division of Psychology, Nottingham Trent UniversityNottinghamUK; ^2^Cambridgeshire and Peterborough NHS Foundation Trust, Addenbrookes HospitalCambridgeUK; ^3^Bamford Centre for Mental Health and Wellbeing, University of UlsterLondonderryUK; ^4^MRC All Ireland Trials Methodology Hub, University of UlsterLondonderryUK; ^1^International Gaming Research Unit, Division of Psychology, Nottingham Trent UniversityNottinghamUK; ^2^Cambridgeshire and Peterborough NHS Foundation Trust, Addenbrookes HospitalCambridgeUK; ^3^Bamford Centre for Mental Health and Wellbeing, University of UlsterLondonderryUK; ^4^MRC All Ireland Trials Methodology Hub, University of UlsterLondonderryUK

**Keywords:** muscle dysmorphia, behavioural addiction, body dysmorphic disorder, body image, obsessive–compulsive disorder, eating disorder

## Abstract

**Background:**

Following the publication of our paper ‘Muscle Dysmorphia: Could it be classified as an addiction to body image?’ in the *Journal of Behavioral Addictions*, two commentaries by Jon Grant and Johanna Nieuwoudt were published in response to our paper.

**Method:**

Using the ‘addiction components model’, our main contention is that muscle dysmorphia (MD) actually comprises a number of different actions and behaviors and that the actual addictive activity is the *maintaining of body image* via a number of different activities such as bodybuilding, exercise, eating certain foods, taking specific drugs (e.g., anabolic steroids), shopping for certain foods, food supplements, and purchase or use of physical exercise accessories. This paper briefly responds to these two commentaries.

**Results:**

While our hypothesized specifics relating to each addiction component sometimes lack empirical support (as noted explicitly by both Nieuwoudt and Grant), we still believe that our main thesis (that almost all the thoughts and behaviors of those with MD revolve around the maintenance of body image) is something that could be empirically tested in future research by those who already work in the area.

**Conclusions:**

We hope that the ‘Addiction to Body Image’ model we proposed provides a new framework for carrying out work in both empirical and clinical settings. The idea that MD could potentially be classed as an addiction cannot be negated on theoretical grounds as many people in the addiction field are turning their attention to research in new areas of behavioral addiction.

When we first thought about writing a paper arguing that muscle dysmorphia (MD) could possibly be classified as an addiction, and more specifically that it was an ‘addiction to body image’ [ABI] (Foster, Shorter & Griffiths, 2015), we knew that the idea might be controversial, particularly to those who have been researching in the field for many years. This is one of the reasons that the editor of the *Journal of Behavioral Addictions* placed our paper in the ‘Debate’ section of the journal. The editor asked us for a list of names of key researchers in the MD field to send the paper to for comment and reaction. For whatever reason, most of those who were given the invitation decided not to respond to our paper but we are very grateful that Johanna Nieuwoudt and Jon Grant took the time to read and comment on what we had written. This paper provides a brief response to some of the issues raised by both Nieuwoudt and Grant (Grant, 2015; Nieuwoudt, 2015).

We agree with Nieuwoudt that there is no agreement as to the specific meanings of terms such as ‘addiction’, ‘behavioral addiction’ and ‘body image’ and that these may all have different meanings among different populations and cultures. However, we operationally defined what we meant by these terms and hope that anyone reading our paper can see how and why we argue that muscle dysmorphia could be associated with the term in the context provided (even if they fundamentally disagree with our speculations). Our main contention is that MD actually comprises a number of different actions and behaviors and that the actual addictive activity is the *maintaining of body image* via a number of different activities such as bodybuilding, exercise, eating certain foods, taking specific drugs (e.g., anabolic steroids), shopping for certain foods, food supplements, and purchase or use of physical exercise accessories.

As Nieuwoudt points out, in the current DSM-5 (American Psychiatric Association, 2013) there is only one behavioral addiction (i.e., ‘gambling disorder’, formerly pathological gambling) that has been given official diagnostic criteria (although another behavioral addiction – ‘internet gaming disorder’ was given diagnostic criteria in Section 3 – ‘Emerging Measures and Models’). The implications of defining potentially problematic behaviors such as gambling or video gaming as genuine behavioral addictions means there is no theoretical reason why other potentially problematic behaviors that do not involve the ingestion of a psychoactive substance (e.g., sex, exercise, work, internet use) cannot be also conceptualized and classified as genuine behavioral addictions if and when the evidence based is considered sufficiently developed to support these conclusions.

Nieuwoudt also notes there is no formal treatment for MD and practitioners in the field have borrowed treatments from related disorders such as body dysmorphic disorder (BDD), eating disorders, and obsessive-compulsive disorders to treat MD. We see no reason why MD could not be treated with therapies used in the treatment of more traditional addictive behaviors such as cognitive-behavioral therapy (CBT) (particularly as our ABI model contains a large cognitive component in that the addiction is maintained by erroneous core beliefs about their own body image). However, as Grant (2015) points out in his commentary of our paper, treatment for MD has (to date) largely utilized pharmacotherapy (selective serotonin reuptake inhibitors) and CBT where both types of treatment have involved uncontrolled case series and reports (Pope et al., 2000). These treatment options are shared with other psychiatric conditions. In part they seem reflective of nosological confusion surrounding MD and suggest that MD (like many addictions) are (at least in part) anxiety-related.

This model is speculative using the addictions component model (Griffiths, 2005) as its theoretical basis. After reading many papers on MD, we were struck by how much of the outward MD behavior described appeared to have similarities to other behavioral addictions. Many of the behaviors associated with MD (e.g., anabolic steroid use, excessive exercise, shopping for specific foods) can be addictive in their own right but we believe these are secondary activities that serve a primary purpose (i.e., maintain body image) that in some people can be operationalized as an addiction and lead to the diagnosis of MD. Nieuwoudt notes that when it comes to the addictions components, there appears to be some support for tolerance and withdrawal as criteria. However, she points out key areas where evidence is not yet present – the evidence only supports extreme anxiety from the missed work-out sessions, and not the other symptoms such as depression, nausea, irritability and stomach cramps. This suggests the need for research that describes the phenomenology of the condition for the user (and through the use of qualitative research in particular).

Using the work of Karim and Chaudhri (2012), Nieuwoudt notes that it may be that the symptoms associated with behavioral addictions are merely symptoms of other disorders. While our hypothesized specifics relating to each addiction component sometimes lack empirical support (as noted explicitly by both Nieuwoudt and Grant), we still believe that our main thesis (that almost all the thoughts and behaviors of those with MD revolve around the maintenance of body image) is something that could be empirically tested in future research by those who already work in the area (something that Grant suggests should happen before *“reclassifying”* MD as an addiction). As Grant (2015) notes:

... we might want to explore the idea that obsessions about body image might reflect a heterogeneous pathophysiology. Some individuals with muscle dysmorphia might be more similar to those with addictions, while others might be more similar to those with obsessive compulsive disorder or body dysmorphic disorder. The notion of muscle dysmoprhia as an addiction, although heuristically appealing, remains speculative and requires additional studies to examine its validity and appropriateness.

Such speculations could be empirically examined. For instance, a study could explore the patterns of symptom presentation in a substantial cohort of patients determining whether there are subtypes of MD symptom expression, and explore how this changes over time (including following treatment). Statistical advances using techniques such as latent class analysis (e.g. Smith, Farrell, Bunting, Houston & Shevlin, 2011) or longitudinal extensions such as latent growth modeling (e.g., Jung & Wickrama, 2008) may help empirically explore this nosological possibility.

Nieuwoudt notes an individual's body image and body dissatisfaction is a key feature in related disorders such as body dysmorphic disorder (Didie, Kuniega-Pietrzak & Phillips, 2010) and eating disorders (Hrabosky et al., 2009; Rosen & Ramirez, 1998). However, in BDD the problem is typically associated with a particular body part rather than the total body image. Interestingly, while MD (‘bigorexia’) is often seen as anorexia nervosa in reverse (in that anorexics feel they are too fat and those with MD feel they are too thin and scrawny), it may be that the ABI model we proposed could equally be applied to some individuals with eating disorders (in that they may engage only in behaviors that they believe stop them from getting fat including starvation and exercise). This is something that Grant acknowledges and notes that our model may be applicable to other disorders (e.g., other compulsive behaviors). Nieuwoudt also pointed out that there has been research by Olivardia, Pope and Hudson (2000) reporting that a large minority of MD sufferers had excellent *“insight”* into their condition. Insight may refer to insight into their illness or their body image concerns, but the knowledge of one’s condition does not necessarily help in alleviating cognitive dysfunction of any addictive behavior.

We were pleased to see that Nieuwoudt (like us) believes that a negative perception of body image has the potential to become an all-consuming and damaging obsession. However, our intention is not to pathologize body image itself. For those affected, the ABI model pathologizes the maintaining behaviors (e.g., excessive exercise, steroid abuse) not body image itself. More specifically, it is the cognitions surrounding addiction in achieving a certain, potentially unrealistic body image that is problematic, not body image itself.

We were also pleased to see that Grant (2015) thought our paper was a *“compelling argument”* for viewing MD as an addiction. We also agree that by examining MD as a potential addiction, our paper provides *“a more provocative look at the possible similarities between obsessional problems and addictions”.* We certainly adhere to the more general thesis that whether a behavior is categorized as obsessive-compulsive or addictive, the elimination of negative feeling by engaging in the behavior is reinforcing (i.e., rewarding) to the individual. We suspect the nature of MD will be fluid throughout the course of the illness. Our speculative model demonstrates what we feel are the more acute stages of MD and further research (both psychological and neurobiological) is needed to further understand the initial stages of MD and how it develops. A neuropsychological approach might highlight shared neural pathways with other disorders to shed more light on the causes of the condition. Like Grant, we believe that such research would help in advancing strategies for both prevention and treatment for MD and other body image obsessions.

We hope that the ABI model we proposed provides a new framework for carrying out work in both empirical and clinical settings. We acknowledge that the model is speculative, provocative, and potentially controversial. The idea that muscle dysmorphia could potentially be classed as an addiction cannot be negated on theoretical grounds; particularly since many people in the addiction field are turning their attention to research in new areas of behavioral addiction. Gambling addiction may well be the ‘breakthrough’ addiction that leads to many other problematic behaviors entering psychiatric diagnostic manuals in the years to come. We are not saying (at this stage) that MD should be included but there are enough similarities between MD and other behavioral addictions that both epidemiological and clinical researchers should at least consider it a possibility and determine it worthy of further investigation.

